# A 6-Month clinical practice pilot study of sucroferric oxyhydroxide on nutritional status in patients on peritoneal dialysis

**DOI:** 10.1186/s12882-022-02878-5

**Published:** 2022-07-09

**Authors:** Luis Perez, Zhiying You, Isaac Teitelbaum, Emily S Andrews, Rachael Reddin, Lorena Ramirez-Renteria, Gabriela Wilson, Jessica Kendrick

**Affiliations:** Division of Renal Diseases and Hypertension, University of Colorado Anschutz Medical Campus, Aurora, CO 80045 USA

**Keywords:** Dialysis, Peritoneal, Appetite, Phosphorus, Binders, Albumin

## Abstract

**Background:**

Hyperphosphatemia is common in patients on peritoneal dialysis (PD). Restricting dietary phosphorus often leads to a decrease in protein intake, which may result in hypoalbuminemia. The high pill burden of phosphate binders may also contribute to compromised appetite and dietary intake. Hypoalbuminemia is associated with an increased risk of morbidity and mortality in PD patients. The goal of this study was to determine if sucroferric oxyhydroxide improves albumin and self-reported measures of appetite in PD patients.

**Methods:**

We performed a prospective, open-label, 6-month, pilot study of 17 adult PD patients from the Denver Metro Area. Patients had to use automated peritoneal dialysis for ≥ 3 months, have a serum albumin ≤ 3.8 g/dL, and have serum phosphate ≥ 5.5 mg/dL or ≤ 5.5 mg/dL on a binder other than SO. SO was titrated to a goal serum phosphate of < 5.5 mg/dL. The primary outcome was change in serum phosphate, albumin, and phosphorus-attuned albumin (defined as albumin divided by phosphorus) over 6 months.

**Results:**

The mean (SD) age and dialysis vintage was 55 ± 13 years and 3.8 ± 2.7 years, respectively. Participants’ serum phosphate significantly decreased with fewer phosphate binder pills/day after switching to SO. There was no change in serum albumin, appetite, or dietary intake. However, participants had significant improvements in phosphorus-attuned albumin.

**Conclusion:**

The transition to SO improved phosphorus control, phosphorus-attuned albumin, and pill burden. There were no significant changes in self-reported appetite or dietary intake during the study. These findings suggest that PD patients maintained nutritional status with SO therapy.

**Trial registration:**

First registered at ClinicalTrials.gov (NCT04046263) on 06/08/2019.

## Background

Electrolyte disorders are common among patients with ESKD on dialysis. Some estimates have historically put the prevalence of hyperphosphatemia (serum phosphate ≥ 5.0 mg/dL) at around 70% of hemodialysis patients, while hyperphosphatemia (serum phosphate ≥ 5.6 mg/dL) on peritoneal dialysis (PD) was recently estimated as high as 35% of patients [1]. Notably, large proportions of these patients had serum phosphorus levels ≥ 6.5 mg/dL [1]. These elevated serum phosphate levels are associated with an increased risk of death and cardiovascular disease in dialysis patients [2–4]. Disordered mineral metabolism is also associated with inflammation in patients with ESKD [5, 6]. Dietary restriction of phosphorus is one crucial component to phosphate control among patients with ESKD. However, dietary restriction alone is not usually successful in controlling serum phosphate levels and is often coupled to phosphate binder therapy. Another concern with dietary phosphorus restriction is that many foods that are high in phosphorus are also high in protein, which further complicates phosphorus control and maintaining adequate protein intake.

Inadequate protein intake and inflammation are two factors that can contribute to low serum albumin [7]. Hypoalbuminemia, as well as malnutrition, are associated with an increased risk of morbidity and mortality in dialysis patients [7, 8]. Many dialysis patients are commonly prescribed high protein diets and dietary supplements, yet serum albumin often remains low or continues to fall. A high protein diet may also result in higher serum phosphate levels and the need for phosphate binders, contributing to pill burden [7]. Sucroferric oxyhydroxide (SO) is an iron-based chewable phosphate binder with a lower pill burden for treatment of hyperphosphatemia in both hemodialysis and PD patients [9–11]. In a retrospective study of 258 PD patients, SO resulted in 74% of patients achieving in-range serum phosphate levels after 6 months and decreased pill burden by 57% [9]. Serum albumin also significantly increased in hypoalbuminemic hemodialysis patients with SO [11]. In PD patients, there was a slight decrease in albumin levels over 6 months, but an increase in phosphorus-attuned albumin and phosphorus-attuned normalized protein catabolic rate [10]. This suggested that SO could improve nutrition status in PD patients. A proposed hypothesis is that improved phosphorus control and reduced pill burden may allow for less dietary restrictions and increased protein intake. We performed a prospective, open-label, pilot study in 17 patients with ESKD on PD to test the hypothesis that management of hyperphosphatemia with SO improves albumin and self-reported measures of appetite.

## Materials and methods

### Study population

Participants were enrolled between January 2020 and February 2021 at the University of Colorado Anschutz Medical Campus. The study eligibility criteria included participants with age ≥ 18 years, on peritoneal (automated) dialysis at least 3 months; treatment Kt/V of ≥ 1.7; serum phosphate ≥ 5.5 mg/dL or ≤ 5.5 mg/dL on a binder other than SO; serum albumin ≤ 3.8 g/dL; able to provide consent; and ability to complete self-reported questionnaires. Participants were excluded for inadequate dialysis treatment, current use of SO; significant comorbid conditions with life expectancy less than 6 months; active malignancy; recent episodes of peritonitis; pregnancy or planning to become pregnant; anticipated kidney transplantation within 6 months; or known adverse side effects to SO. All participants provided written informed consent before study entry. The study protocol and informed written consent were approved by the Colorado Multiple Institutional Review Board (Aurora, CO) and was first registered at ClinicalTrials.gov (NCT04046263) on 06/08/2019. All procedures performed were in accordance with the ethical standards of the 1964 Helsinki Declaration and its later amendments. Informed consent was obtained from the subjects as specified in the ICMJE Recommendations.

### Study design

The study was a 6-month prospective, open-label, pilot study. Participants who met eligibility criteria and provided informed consent underwent screening and began a 2-week washout period if previously on a phosphate binder. Following baseline testing, participants were started on SO and underwent follow-up testing at months 3 and 6 and monthly checks for routine laboratory values, adverse events, and compliance. No additional dietary counseling, other than standard of care practice, was provided to participants during the study. Laboratory and clinical treatment data were obtained from participants treatment records throughout the course of the study. Clinical blood laboratory values were collected from standard of care blood draws every 3 months. The PD treatment schedules and parameters were consistent over the course of the study.

### Sucroferric oxyhydroxide dosing

Participants were started on 1 tablet (500 mg) SO 3 times daily with meals. The SO dosage was titrated monthly in increments of 500 mg (1 tablet) per day until serum phosphate was < 5.5 mg/dL.

### Study outcomes

The primary study endpoints were change in serum phosphorus and serum albumin, and phosphorus-attuned albumin. Secondary endpoints included changes in appetite. Phosphorus-attuned albumin was defined and calculated by dividing serum albumin by serum phosphorus levels (i.e. adjustment, units of 10x^3^) [9]. Adjustment of albumin with serum phosphorus aims to evaluate if lower protein intake contributed to lower serum phosphorus and/or albumin levels, which is a potential concern for malnutrition and mortality [9–11]. In this regard, ideally patients reduce serum phosphorus levels while maintaining or increasing albumin represented by increased in phosphorus-attuned albumin [9–11]. Phosphate binder pill burden and pill compliance were calculated based off phosphate binder prescription upon enrollment, during the study, and actual reported or accounted for pill consumption. Participants completed the Appetite and Diet Assessment Tool (ADAT) and 3-day dietary records at each of the three major study time-points [12]. Research staff provided instructions for patients to record dietary history and dietary records were entered into the National Institutes of Health Automated Self-Administered 24-h assessment tool (ASA-24) for nutrient analysis. Participants also completed a gastrointestinal symptom and side effects questionnaire each month.

### Statistical analysis

Descriptive statistics were used to summarize all data. For example, mean ± SD or median and interquartile range (IQR) were calculated for a continuous variable if appropriate, and frequency and proportion were reported for a categorical or ordinal variable. Data transformation was performed before further analysis if appropriate. The primary analyses consisted of repeated measures data analysis with mixed effects model for a continuous outcome and with generalized estimating equations for a categorical or ordinal outcome. All participants with at least one measure on an outcome variable were included in analysis. The paired t-test was used in the pre-post comparison of pill count. The two-sided significance level of 0.05 was used in making a conclusion. All analyses were performed using SAS software, version 9.4 (SAS Institute Inc.).

## Results

### Study participants

Seventeen patients were enrolled in the study and 12 patients completed the entire study. One patient completed the study early due to receiving a kidney transplant. Baseline characteristics are shown in Table 1. Participants had a mean age of 55 ± 14 years. The mean dialysis vintage was 3.6 ± 2.5 years. The majority of patients were male (65%) and White/Caucasian (82%). Six individuals (35%) identified as Hispanic/Latino. Eighty-eight percent of patients were on a phosphate binder at baseline and the majority were on sevelamer or a combination of sevelamer and calcium-based binders (Table 1). Median baseline phosphate binder burden prior to washout was 12.0 (9.0 – 15.0) pills/day. Average compliance for SO over the course of the study was 73.9 ± 24.5%.

**Table 1:** Participant demographics at baseline

Characteristic	Values
Age, years	55 ± 14
Sex, N (%)	
Male	11 (65%)
Female	6 (35%)
Race, N (%)	
Non-Hispanic white	14 (85%)
Hispanic	6 (35%)
Weight, kg	85.2 ± 21.9
BMI, kg/m^2^	28.9 ± 5.7
Dialysis vintage, years	3.6 ± 2.5
Diabetes, N (%)	8 (47%)
Prescribed PB, N (%)	15 (88%)
Calcium acetate, N (%)	2 (11.8%)
Sevelamer, N (%)	9 (52.9%)
Sevelamer + calcium-based binder, N (%)	4 (23.5%)

*BMI* Body mass index, *PB* Phosphate binder, All values are mean (SD) unless otherwise specified

### Effects of SO on serum phosphorus and albumin

**Fig. 1:**
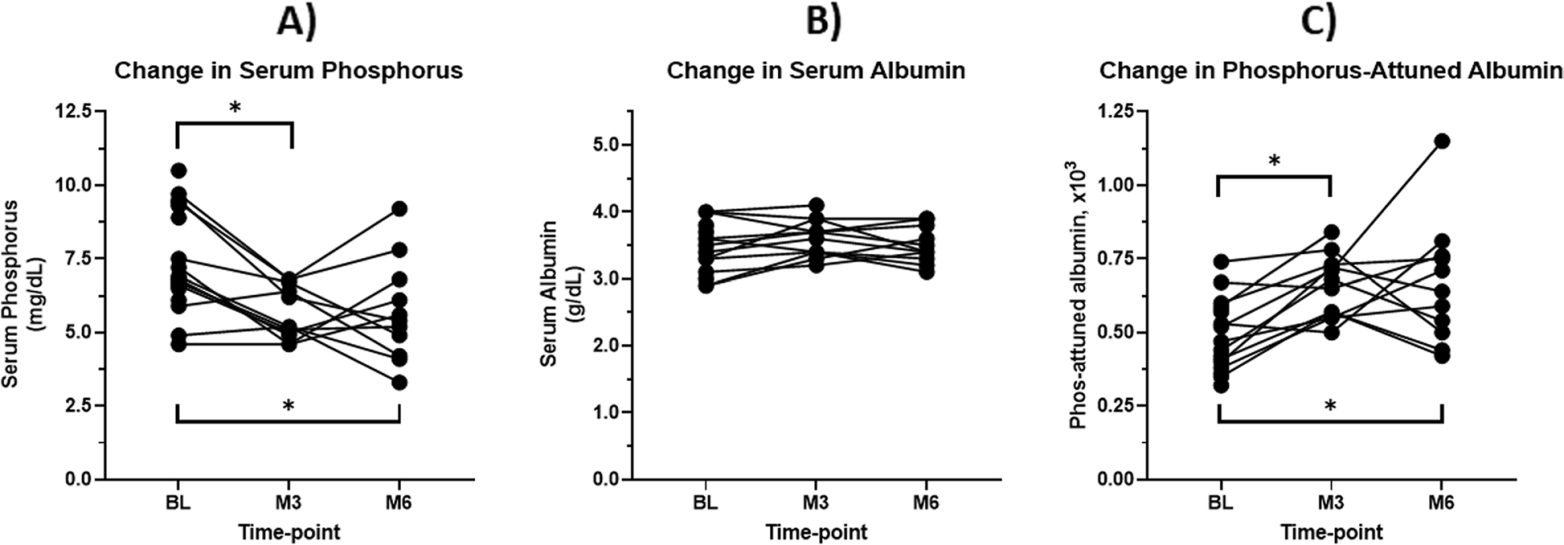
Changes in Study Clinical Values. **A** Change in Serum Phosphorus. **B** Change in Serum Albumin. **C** Change in Phosphorus Attuned Albumin. BL, baseline visit; M3, month-3 visit; M6, month-6 visit. * *P* value < 0.05

Participants had a significant reduction in serum phosphorus at both 3 months and 6 months, when compared to baseline (all *p* < 0.01, Table 2). Approximately 55% of patients achieved a serum phosphorus level of < 5.5 mg/dL at month 6. However, there were no significant changes in serum albumin at any time-point (*p* > 0.05). There was a significant increase in phosphorus-attuned albumin, driven by an increase from baseline to month 3 that was maintained at month 6 (all *p* < 0.05). Changes in serum phosphorus, albumin, and phosphorus-attuned albumin are depicted in Fig. 1. There were no significant changes in parathyroid hormone (PTH) levels with SO use (*p* > 0.05). Phosphate pill burden significantly decreased from a median of 12 (10.5 – 13.0) pills/day before SO to 5.0 (3.0 – 6.0) pills/day with SO (*p* < 0.01) for subjects who completed the study.

**Table 2:** Changes in study outcomes

		**Time-point**		
**Variable**	**Baseline (BL)**	**3 Month (M3)**	**6 Month (M6)**	***P*-Value**
**All participants (n)**	*n* = 17	*n* = 12	*n* = 11^b^	
Phosphorus, mg/dL^a^	7.47 ± 1.76	5.63 ± 0.88	5.69 ± 1.72	< 0.001
Albumin, g/dL	3.49 ± 0.37	3.61 ± 0.27	3.50 ± 0.27	0.25
Phosphorus-attuned albumin, x10^3a^	0.49 ± 0.12	0.65 ± 0.10	0.67 ± 0.21	< 0.05
Calcium, mg/dL^a^	8.22 ± 1.03	8.83 ± 0.84	8.63 ± 1.13	< 0.001
Intact PTH, pg/mL	416 (292–631)	382 (128–578)	361 (237–540)	0.33
**Completed participants (*n* = 12)**				
Phosphorus, mg/dL^a^	7.17 ± 1.76	5.69 ± 0.89	5.69 ± 1.72	< 0.001
Albumin, g/dL	3.42 ± 0.38	3.56 ± 0.24	3.5 ± 0.27	0.21
Phosphorus-attuned albumin, x10^3a^	0.50 ± 0.13	0.64 ± 0.09	0.67 ± 0.21	0.02
Calcium, mg/dL^a^	8.13 ± 1.17	8.75 ± 0.83	8.63 ± 1.13	< 0.01
Intact PTH, pg/mL	340 (166–462)	377 (128–578)	361 (237–540)	0.37
**Dietary Intake**				
Calories	1621 ± 648	1383 ± 868	1575 ± 567	0.42
Calories/kg	20.9 ± 11.1	18.1 ± 13.6	20.2 ± 9.0	0.53
Protein, g	77 ± 35	62 ± 38	74 ± 39	0.16
Protein, g/kg	1.0 ± 0.6	0.8 ± 0.6	1.0 ± 0.6	0.21
Phosphorus, mg	1159 ± 525	973 ± 685	1097 ± 419	0.36

All values are presented as mean ± standard deviation, except PTH is reported as median (interquartile range). PTH, parathyroid hormone. ^a^ BL different from 3 and 6 M (*P* value < 0.05). ^b^ One subject not accounted for (*n* = 12) was an early end of study visit

### Effects of SO on appetite

There did not appear to be any changes in appetite or desire to change diet with SO (*p* > 0.05, Table 3). There were also no significant changes in self-reported appetite within the ADAT (all *p* > 0.05), except for an increase in desire to change diet at month 3 (*p* = 0.037), which was no longer present at month 6, when compared to baseline (*p* > 0.05, Table 3).

**Table 3:** Changes in appetite

		**Time-point (count, %)**		
**Variable**	**Baseline (BL)**	**3 Month (M3)**	**6 Month (M6)**	***P*-Value**
Appetite level				0.44
Very good	4 (24%)	1 (8%)	0 (0%)	
Good	6 (35%)	7 (58%)	6 (35%)	
Fair	6 (35%)	2 (17%)	4 (24%)	
Poor	1 (6%)	2 (17%)	1 (6%)	
Appetite change				0.95
No	14 (82%)	10 (83%)	9 (53%)	
Yes	3 (18%)	2 (17%)	2 (12%)	
Desire to change diet				0.14
No	9 (53%)	10 (83%)	6 (35%)	
Yes	8 (47%)	2 (17%)	5(29%)	

Month 6 data does not total to 100% due subjects that did not complete missing measures

At baseline, 41% of participants self-reported difficulty following their diet. The five major reasons for difficulty following their diet included: “Do not feel like the foods I am supposed to eat” (12%), “Do not feel like cooking” (10%), “Do not feel like preparing food” (6%), “Do not understand what I am supposed to eat” (4%), and “Do not have control over my food choices” (4%). The majority of open-ended responses for participants wanting to change diet generally fell into the category of desire to liberalize their diet for more freedom in food choices. Notably, 83% of participants reported going out to eat throughout the course of the study, with a mean frequency of 3.7 ± 2.9 times per month. Methods of eating out included 57% fast food, 41% restaurant, 14% friend/relatives, and 8% cafeteria. At home, greater than 90% of participants reported having a stove, refrigerator, freezer, microwave oven, and food processor/blender (60% reported having a toaster oven).

### Side effects and symptoms

There were no serious adverse events over the course of the study related to the study drug. Twelve patients (71%) completed the study. Two patients withdrew due to side effects (diarrhea), 1 patient changed to hemodialysis and 2 patients died (unrelated to the study). One patient had peritonitis following a hospitalization for COVID-19 infection. Participants did not require reduction of study medications during the study, except for one subject that subsequently withdrew from participation. At baseline, 94% (*n* = 17) of participants had reported experiencing existing gastrointestinal symptoms. This decreased to 82% (*n* = 9) of participants by month 6 of the study (Table 4). For those who reported gastrointestinal symptoms, the overall reported frequency count (present vs. not present) of each over the whole course of the study was 49% (*n* = 34) for diarrhea, 44% for constipation (*n* = 31), 28% for bloating (*n* = 19), 26% (*n* = 18) for nausea, 25% (*n* = 17) for vomiting (Table 4). Notably, there were seven self-reported cases of “severe” gastrointestinal symptoms reported (*n* = 2 diarrhea, *n* = 3 constipation, and *n* = 2 bloating), including two that were already present at baseline (*n* = 1 constipation and *n* = 1 bloating). Subjects attributed these symptom events to their non-study drug medications approximately 19% (*n* = 13) of the time. Significantly fewer patients reported bloating with SO. For other self-reported side effects over the 6-month course of the study, participants reported 84% (*n* = 56) black/tarry/or bloody stools, 25% (*n* = 17) color changes in stools, and 4% (*n* = 3) rashes. However, these self-reported symptoms numerically decreased in frequency over the course of the study.

**Table 4:** Gastrointestinal symptoms

Symptom Frequency N (%)	Baseline	Month 6
GI symptoms experienced	16 (94%)	9 (82%)
Nausea	3 (19%)	2 (13%)
Vomiting	3 (19%)	2 (13%)
Bloating	9 (56%)	0 (0%)
Diarrhea	6 (38%)	4 (25%)
Constipation	10 (63%)	5 (31%)

*GI* Gastrointestinal

## Discussion

In this open-label, prospective, pilot trial, we did not find that sucroferric oxyhydroxide independently improved serum albumin or appetite in patients with ESKD on PD. Consequently, nutrient intake did not appear to change over the course of the study. However, patients did significantly reduce their serum phosphorus levels and pill burden during the study. Additionally, study participants did have significant increases in phosphorus-attuned albumin. This suggested that participants may have maintained appetite or potentially attenuated any potential reductions in appetite. There were no changes in PTH at any study time-point. Sucroferric oxyhydroxide was well-tolerated without any significant adverse events. To our knowledge, this is the first study prospectively examining the effects of sucroferric oxyhydroxide therapy on albumin, appetite, and dietary changes among patients on PD.

While we didn’t find a significant increase in serum albumin, we did find an increase in phosphorus-attuned albumin. The increase in phosphorus-attuned albumin suggests an overall improvement in the nutritional status of the participants. Additionally, it suggests that the decreases in serum phosphorus were the result of SO, not decreases in protein intake. We confirmed this with the nutritional information collected which did not show a significant change in dietary protein intake. Our findings are similar to a recent retrospective database analysis [9]. PD patients prescribed SO as part of routine care had significant reductions in serum phosphorus and pill burden. While the authors found a slight decrease in serum albumin over the 6 month follow-up period with SO, phosphorus-attuned albumin significantly increased similar to our findings. While this metric has not been used routinely in dialysis patient care, it is important that any rise in albumin should not be associated with a concurrent rise in phosphorus.

Similar results have also been found in patients with ESKD on hemodialysis. In a one-year cohort analysis, we found that HD patients who switched from non-sucroferric oxyhydroxide based binders to sucroferric oxyhydroxide also had significant reductions in serum phosphorus levels and pill burden [9], as observed in this current study of PD patients. We also found improvements in phosphorus-attuned albumin with no change in dietary intake comparable to previous studies [9, 10]. Kalantar-Zadeh et al. [10] found sucroferric oxyhydroxide therapy was associated with reductions in serum phosphorus levels and pill burden in both hypoalbuminemic and non-hypoalbuminemic HD patients. Serum albumin levels increased significantly in the hypoalbuminemic HD patients but serum albumin did not change in the non-hypoalbuminemic patients. These studies suggest that SO may improve the nutritional status of patients. However, these studies were retrospective and observational and to date, no prospective trials have been done in HD patients.

Previous studies have reported on the high prevalence of poor appetite in PD patients [13–15], which was also observed in our study. Reduced appetite predicts inadequate nutrition and malnutrition in PD patients [14]. Both reduced appetite and malnutrition are associated with increased mortality among patients on PD [1]. Low-albumin is also associated with mortality in patients on dialysis [16]. However, hypoalbuminemia may be the result of both malnutrition and inflammation or other processes [17, 18]. Unfortunately, we were unable to obtain biochemical measures of inflammation (C-reactive protein and interleukin-6) and other markers (fibroblast growth factor 23 and prealbumin) in the majority of study participants at all time-points due to the COVID-19 pandemic. PD solution-induced hypophagia has been one proposed mechanism [13] that may have contributed to reported decreases in this study. However, studies have not widely quantified this effect and we did not account for this in the current study. Additionally, the presence of dialysate in the peritoneal cavity [13, 15] may have potentially contributed to delayed gastric emptying and reduced intestinal motility and subsequently decreased appetite. In our study, 8 participants had day dwells, but their prescription remained stable throughout the study. Other hypotheses for poor appetite include phosphate binder pill burden, pre-existing gastrointestinal symptoms, and gastrointestinal side effects as a result of phosphate binders.

In the current study, we found that participants’ appetite increased at 3 months and decreased at 6 months, while caloric and protein intake decreased at 3 months and increased at 6 months. However, the categorical and numerical trend was for poorer intake over the whole study duration. This indicates that patients self-reported appetite may not have been reflective of the nutrient intakes observed in this study. Notably, Wright et al. [19] reported that PD patients appear to normalize their self-reported appetite at lower levels than healthy controls. PD patients in the Wright et al. [19] study also report lower dietary nutrient intakes than healthy controls. Nutrient intakes observed in the current study were generally in-line with previous research studies, yet did not meet nutritional recommendations for PD.

We found that SO was well-tolerated even at the average final dose of 5 tablets per day. Previous trials in PD and hemodialysis patients found an average dose of 4 tablets per day of SO was well-tolerated and effective over a one-year time period [20]. In this larger study, withdrawal from SO from adverse events occurred in only 8.2% of participants. Recent real-world studies of SO have also shown that it is effective and well-tolerated [21]. We did not obtain information beyond the 6-month time period in our study, so we do not know if patients were able to continue on SO and thus do not have longer data regarding effectiveness or side effects.

Our study does have several limitations, including the lack of a control group and the small sample size. With the small sample size we were unable to examine subgroups including subgroups based on initial baseline phosphate binders (e.g. sevelamer vs. calcium acetate). The COVID-19 pandemic also significantly impacted our study. Due to the pandemic, we were unable to have serum phosphorus and albumin levels drawn at the same time of day in all participants. We recognize that phosphorus levels may change before or after meals. However, a recent study found that a high phosphorus meal only has a negligible effect on plasma phosphate compared to low phosphorus meal in PD patients [22]. Additionally, we did not have information on D/P creatinine ratio at baseline and end of study in all participants. We acknowledge changes in D/P creatinine ratio may affect serum albumin and may change over an extended period of time, however given the short time frame of this study it is unlikely there were significant changes in D/P creatinine.

## Conclusion

In conclusion, 6 months of sucroferric oxyhydroxide therapy did not improve appetite or serum albumin in patients on PD, but did significantly reduce both serum phosphorus and phosphorus binder pill burden. Furthermore, participants had significant increases in phosphorus-attuned albumin, suggesting that reductions in phosphorus were not due to limitations of protein intake or other significant nutritional changes. Future multi-center trials should investigate if reduced pill burden from sucroferric oxyhydroxide can independently improve appetite and dietary intake, resulting in improvements in markers of nutritional status. Furthermore, studies should investigate the impact of sucroferric oxyhydroxide on inflammation and patient-related outcomes such as quality of life.

## Data Availability

The datasets used and/or analyzed during the current study are available from the corresponding author on reasonable request.
